# Myocardial oxygen handling and metabolic function of ex-situ perfused human hearts from circulatory death donors

**DOI:** 10.1016/j.jhlto.2024.100159

**Published:** 2024-09-26

**Authors:** Jorik H. Amesz, Sanne J.J. Langmuur, Mark F.A. Bierhuizen, Dwight Dumay, Pieter C. van de Woestijne, Jelena Sjatskig, Lisa E. Sluijter, Dirk J. Duncker, Olivier C. Manintveld, Yannick J.H.J. Taverne

**Affiliations:** a Translational Cardiothoracic Surgery Research Lab, Department of Cardiothoracic Surgery, Erasmus University Medical Center, Rotterdam, the Netherlands; b Department of Cardiothoracic Surgery, Erasmus University Medical Center, Rotterdam, the Netherlands; c Erasmus MC Transplant Institute, Erasmus University Medical Center, Rotterdam, the Netherlands; d Department of Cardiology, Erasmus University Medical Center, Rotterdam, the Netherlands; e Clinical Perfusion, Department of Cardiothoracic Surgery, Erasmus University Medical Center, Rotterdam, the Netherlands; f Division of Experimental Cardiology, Department of Cardiology, Erasmus MC Cardiovascular Institute, Erasmus University Medical Center, Rotterdam, the Netherlands

**Keywords:** cardiac transplantation, ex-situ heart perfusion, donation after circulatory death, myocardial oxygen consumption, normothermic machine perfusion

## Abstract

**Background:**

This study investigated oxygen handling of human hearts donated after circulatory death (DCD) on normothermic ex-situ heart perfusion (ESHP) and evaluated oxygen handling markers as adjuncts to cardiac viability assessment.

**Methods:**

This single-center retrospective study included human DCD heart transplantation procedures using ESHP. Lactate concentrations, blood gas, myocardial oxygen consumption (MVO_2_), delivery (MDO_2_), and extraction (MEO_2_), coronary blood flow (CBF), coronary vascular resistance (CVR), and adenosine infusion were reported over time. Correlation between parameters was assessed, and statistical testing compared patients who did and did not require extracorporeal membrane oxygenation (ECMO) support after transplantation.

**Results:**

Lactate concentrations decreased during ESHP in all transplanted hearts (*n* = 25) and increased in 1 rejected heart. Arterial partial pressure of oxygen (*P*O_2_) was 75.2 ± 2.9 kPa, with an arteriovenous Δ*P*O_2_ of 44.8 ± 10.4 kPa. Oxygen saturation was 100% in most arterial and venous samples. Average MVO_2_ was 2.7 ± 0.6 ml/min/100 g myocardium, MDO_2_ 98.5 ± 20.4 ml/min, and MEO_2_ 8.6 ± 1.8%. Average CVR was 0.025 ± 0.006 mm Hg min/ml/100 g and increased over time. Δ*P*O_2_ correlated strongly with MVO_2_ (R = 0.797, *p* < 0.001) and lactate trend (R = 0.799, *p* < 0.001) in transplanted hearts, without differences compared to the rejected heart with increasing lactate. Adenosine infusion on ESHP was significantly higher in patients requiring ECMO post-transplantation vs non-ECMO cases (11.7 (4.5-21.0) vs 2.2 (1.5-6.7) ml/h, *p* = 0.039).

**Conclusions:**

Hearts on normothermic ESHP receive excessive MDO_2_, due to high *P*O_2_ and CBF, while the MVO_2_ is relatively low. Thus, CBF and *P*O_2_ can potentially be lowered. Furthermore, Δ*P*O_2_ could serve as additional marker of metabolic function under these hyperoxic circumstances. The adenosine infusion rate might predict post-transplantation ECMO requirement.

## Background

Normothermic ex-situ heart perfusion (ESHP), in which the heart is preserved in a beating state, has been successful in enlarging the donor pool using brain death (DBD) and circulatory death (DCD) donor hearts.^1^, ^2^, ^3^, ^4^, ^5^, ^6^, ^7^ Nevertheless, DCD hearts are prone to metabolic decline as they experience significant warm ischemia before administration of cardioplegia.^8^ Donor heart assessment before transplantation is essential, especially with the trend toward utilizing more marginal donors which are more vulnerable to ischemia-reperfusion injury.^9^ Currently, this assessment is solely based on a combination of visual contractile inspection, coronary blood flow (CBF), aortic pressure (AoP), and blood lactate trends during ESHP.^10^ However, conflicting results have been published on the predictive value of lactate trends.^11^, ^12^, ^13^, ^14^ Therefore, additional real-time, noninvasive assessment parameters are warranted, especially in cases with increasing lactate profiles and more marginal donors.

Functional parameters, such as ejection fraction, stroke work, and dP/dt, cannot be measured on the only approved clinically available ESHP device (Transmedics Organ Care System [OCS]) due to the unloaded left ventricle.^10^, ^15^ Preclinical data suggest a correlation, albeit moderate, between both myocardial oxygen consumption (MVO_2_) and coronary vascular resistance (CVR) during ESHP, and post-transplant function.^16^, ^17^, ^18^, ^19^ The association between myocardial contractility, CBF, and MVO_2_ is well established.^20^, ^21^ Yet, no study investigated the use of these parameters in a clinical transplantation setting with human donor hearts transported on ESHP. Therefore, the goal of this study was to (1) investigate oxygen handling of DCD hearts on the OCS and (2) determine valid real-time metabolic parameters as adjuncts to graft viability assessment.

## Methods

This is a retrospective study including human DCD hearts transported on ESHP to the Erasmus Medical Center (Rotterdam, The Netherlands) between May 2021 and September 2023.

Hearts were procured from DCD donors in a standard manner by the surgical team and revived on the OCS (OCS Heart, TransMedics Inc., Andover, MA) according to the standard protocol.^22^ Functional warm ischemic time (FWIT) was defined as the period between drop of systolic blood pressure <50 mm Hg and cardioplegic flush of the donor heart. The OCS was primed with 500 ml of priming solution, 125 ml 20% albumin, and 1200 to 1800 ml of blood from the cardiac donor. The aorta was cannulated and mounted on the OCS. The caval veins were closed and a pulmonary artery cannula connected. This allowed the right ventricle to actively pump the coronary sinus return into the pulmonary artery cannula, where CBF was measured. Standard CBF settings were 700 to 800 ml/min with a target AoP of 75 to 80 mm Hg.^22^ Adenosine, a potent vasodilator, was automatically infused. A left vent was placed in the open left atrium to limit left ventricular distension. Arterial and venous blood samples were taken for blood gas analyses, with frequent sampling in the first hour after reperfusion and approximately 1 sample per hour after stabilization.^22^ Electrolytes were supplemented and pH corrected where needed. Temperature was set to 37°C during priming and decreased to 34°C after reperfusion of the heart. Hearts were accepted for transplantation if lactate levels in the blood were decreasing over time and contractility was good.^22^

Patients provided informed consent for anonymous use of their medical details for scientific publication purposes related to DCD transplantation, as approved by the Medical Ethics Committee of the Erasmus MC (MEC 2020-0106) and in compliance with the ISHLT Ethics statement. Sex and body weight were obtained from donor records to estimate heart weight from female (HW_F_) and male (HW_M_) donors (Equations (1) and (2)).^23^, ^24^ Blood gas analyses, AoP, CBF, and adenosine infusion were retrieved from procedure logs.

(1) $${\mathrm{HW}}_{F}\;(g)=2.153\times \mathrm{BW}+104.6$$

(2) $${\mathrm{HW}}_{M}\;(g)=2.492\times \mathrm{BW}+141.2$$

### Blood gas analysis

Arterial and venous blood samples were analyzed using a handheld blood gas analyzer (i-STAT, Abbott Laboratories, Chicago, IL). Partial pressures of oxygen (*P*O_2_) and carbon dioxide (*P*CO_2_), pH, lactate, glucose, hemoglobin concentrations ([Hb]), and oxygen saturation (*S*O_2_) in the arterial and venous samples were measured. Procedures with less than 2 sampling points were excluded from the analysis.

### Myocardial oxygen consumption

Oxygen content in arterial (C_a_O_2_) and venous (C_v_O_2_) blood was calculated based on the concentration of *P*O_2_, [Hb], and *S*O_2_ (Equation (3)). Myocardial oxygen delivery (MDO_2_) was determined by multiplying CBF by C_a_O_2_ (Equation (4A)). MVO_2_ was calculated according to Equation 5(A) with normalization for estimated HW (Equation (5B)).^16^ Myocardial oxygen extraction (MEO_2_) was calculated by dividing MVO_2_ by MDO_2_.

(3) $$\mathrm{Content}\;O_{2}\;(ml\;O_{2}/ml)=\left[\mathrm{Hb}\right]\times 1.34\times {\mathrm{SO}}_{2}+{\mathrm{PO}}_{2}\times 0.0031$$

(4A) $${\mathrm{MDO}}_{2}\;(ml\;O_{2}/\min)=\mathrm{CBF}\times C_{a}O_{2}$$

(4B) $${\mathrm{MDO}}_{2}/\mathrm{HW}\;(ml\;O_{2}/\min/100\;g)=\frac{\mathrm{CBF}\times C_{a}O_{2}}{\mathrm{HW}÷100}$$

(5A) $${\mathrm{MVO}}_{2}\;\left(ml\;O_{2}/\min\right)=\mathrm{CBF}\times \left(C_{a}O_{2}-C_{v}O_{2}\right)$$

(5B) $${\mathrm{MVO}}_{2}/\mathrm{HW}\;\left(ml\;O_{2}/\min\;/100\;g\right)=\frac{\mathrm{CBF}\times \left(C_{a}O_{2}-C_{v}O_{2}\right)}{\mathrm{HW}÷100}$$

### Coronary vascular resistance and conductance

CVR was calculated by dividing the diastolic AoP by CBF with correction for HW (Equation (6B)),^16^ and coronary vascular conductance (CVC) as the inverse (Equation (7B)). The auto AoP mode of the OCS automatically set the delivery rate of adenosine, ranging from 0 to 30 ml/h, until AoP decreased to the target pressure, and could be set manually >30 ml/h. The administration rates of adenosine were reported throughout the procedure and concentration of adenosine was 1.5 mg/liter in the infusate.

(6A) $$\mathrm{CVR}\;\left(\mathrm{mmHg}∙\min/ml\right)=\frac{\mathrm{aortic}\;\mathrm{diastolic}\;\mathrm{pressure}}{\mathrm{CBF}}$$

(6B) $$\mathrm{CVR}/\mathrm{HW}\;\left(\mathrm{mmHg}∙\min/ml/100\;g\right)=\frac{\mathrm{aortic}\;\mathrm{diastolic}\;\mathrm{pressure}}{\left(\mathrm{CBF}÷\mathrm{HW}÷100\right)}$$

(7A) $$\mathrm{CVC}\;(ml/\min/mmHg)=\frac{\mathrm{CBF}}{\mathrm{aortic}\;\mathrm{diastolic}\;\mathrm{pressure}}$$

(7B) $$\mathrm{CVC}/HW\;(ml/\min/100\;g/mmHg)=\frac{\left(\mathrm{CBF}÷\mathrm{HW}÷100\right)}{\mathrm{aortic}\;d\mathrm{iastolic}\;\mathrm{pressure}}$$

### Statistical analysis

Data were tested for normality using density plots, quantile-quantile plots, and a Shapiro-Wilk test. Data were presented as mean ± standard deviation if normally distributed and as median (interquartile range) otherwise. Pearson’s correlation tests were performed to test for correlation between blood gas data, CBF, AoP, and calculated metabolic parameters. A correlation coefficient of <0.3 was considered as weak, ≥0.3 to <0.7 as moderate, and ≥0.7 as strong correlation. An unpaired *t*-test was performed in case of normally distributed data between patients who did and did not require extracorporeal membrane oxygenation (ECMO) post-transplantation and not. A Mann-Whitney U test was performed in case of non-normally distributed data. A 2-tailed *p*-value of ≤0.05 was considered statistically significant.

## Results

Twenty-six DCD hearts were included in the study, of which 25 hearts were transplanted and 1 was rejected due to increasing lactate levels. Blood gas data were available for 19 transplant procedures whereas CVR and lactate concentrations could be retrieved from all procedures.

### Donor and recipient characteristics

Mean donor age from transplanted hearts was 41 ± 11 years. Fifteen (60%) donors were male and the estimated HW was 355 ± 24 g for male and 263 ± 25 g for female donors. Median FWIT was 15 (15-21) minutes. The donor of which the heart was rejected for transplantation was <30 years old.

Median recipient age was 51 (35**-**59) years with 80% male patients. Nineteen of 25 recipient patients (76%) underwent previous cardiac surgery, of whom 14 patients had a left ventricular assist device in-situ and 2 patients underwent a previous heart transplantation.

### Lactate

All transplanted DCD hearts showed decreasing lactate levels over time, with an average arterial decrease of 0.97 ± 0.53 mmol/liter/h, starting from a concentration of 7.24 ± 1.43 mmol/liter (Table 1). The rejected heart showed an upward lactate trend of 1.19 mmol/liter/h, starting from 5.60 mmol/liter ranging up to 8.19 mmol/liter, despite normal visual contractile function and no evidence of cardiac malperfusion.

**Table 1:** Arterial and Venous Lactate (*n* = 25) and Blood Gas Overview (*n* = 19) of DCD Hearts on the OCS That Were Transplanted

	Reperfusion	1 h	2 h	3 h	Trend (mmol/liter/h)
Lactate (mmol/liter)					
− Arterial	7.24 ± 1.43	6.66 ± 1.52	5.43 ± 1.36	4.49 ± 1.54	−0.97 ± 0.53
− Venous	7.13 ± 1.45	6.78 ± 1.04	5.22 ± 1.40	4.38 ± 1.57	−0.95 ± 0.54
					
	Reperfusion	1 h	2 h	3 h	Average
Glucose (mmol/liter)					
− Arterial	12.0 ± 3.1	12.0 ± 3.8	12.8 ± 3.8	13.3 ± 3.0	12.5 ± 3.3
pH					
− Arterial	7.30 ± 0.10	7.38 ± 0.09	7.39 ± 0.07	7.42 ± 0.06	7.38 ± 0.07
− Venous	7.29 ± 0.10	7.36 ± 0.09	7.37 ± 0.07	7.40 ± 0.06	7.36 ± 0.08
*P*O_2_ (kPa)					
− Arterial	74.5 ± 3.3	74.3 ± 4.3	75.2 ± 4.1	75.8 ± 4.5	75.2 ± 2.9
− Venous	30.9 ± 8.7	30.4 ± 9.3	31.3 ± 9.8	29.8 ± 10.3	30.4 ± 9.2
*P*CO_2_ (kPa)					
− Arterial	5.9 ± 1.5	5.7 ± 1.0	5.5 ± 1.0	5.5 ± 1.0	5.6 ± 1.0
− Venous	5.8 ± 1.4	5.5 ± 1.0	5.4 ± 1.0	5.5 ± 1.1	5.5 ± 1.0

DCD, donation after circulatory death; PCO_2_, partial pressure of carbon dioxide; PO_2_, partial pressure of oxygen.

### Blood gas

Upon reperfusion, the pH was slightly acidic (pH: 7.30 ± 0.10), which normalized to physiological levels within the first hour (Table 1). Average [Hb] was 8.2 ± 1.9 g/dl. Arterial glucose measurements showed hyperglycemia with an average glucose concentration of 12.5 ± 3.3 mmol/liter. A supraphysiological C_a_O_2_ was constantly delivered to hearts on ESHP, with an average *P*aO_2_ of 75.2 ± 2.9 kPa (Table 1). Mean arteriovenous difference in *P*O_2_ (Δ*P*O_2_) was 44.8 ± 10.4 kPa. Venous *S*O_2_ levels were 100% in the majority of samples. *P*aCO_2_ (5.6 ± 1.0 kPa) and *P*vCO_2_ (5.5 ± 1.0 kPa) were within the physiological range (Table 1) without significant arteriovenous difference.

### Myocardial oxygen consumption and coronary vascular resistance

Average CBF was 773 ± 52 ml/min and stable over time. Average MDO_2_ was 98.5 ± 20.4 ml/min. MVO_2_ was 8.4 ± 2.5 ml/min with an MEO_2_ of 8.6 ± 1.8% and remained stable throughout the ESHP procedures (Table 2).

**Table 2:** Overview of Myocardial Oxygen Handling Parameters (*n* = 19) and Diastolic AoP, CBF, CVR, and CVC (*n* = 25) of DCD Hearts That Were Transplanted

	Reperfusion	1 h	2 h	3 h	Average
MVO_2_ (ml/min)	8.5 ± 2.9	8.4 ± 2.7	8.1 ± 2.5	8.8 ± 3.7	8.4 ± 2.5
MVO_2_/HW (ml/min/100 g)	2.7 ± 0.8	2.7 ± 0.6	2.6 ± 0.5	2.7 ± 0.8	2.7 ± 0.6
MDO_2_ (ml/min)	101.7 ± 31.0	96.2 ± 25.9	94.4 ± 23.4	91.1 ± 27.0	98.5 ± 20.4
MDO_2_/HW (ml/min/100 g)	32.5 ± 8.7	30.9 ± 6.8	30.5 ± 7.0	29.2 ± 8.9	31.7 ± 6.1
MEO_2_ (%)	8.4 ± 2.9	8.8 ± 1.6	8.5 ± 2.2	8.7 ± 1.2	8.6 ± 1.8
Diastolic AoP (mm Hg)	45 ± 15	58 ± 11	61 ± 10	62 ± 8	59 ± 7
CBF (ml/min)	795 ± 53	804 ± 65	784 ± 73	747 ± 70	773 ± 52
CBF/HW (ml/min/100 g)	256 ± 46	259 ± 44	253 ± 48	242 ± 54	250 ± 46
CVR (mm Hg min/ml)	0.058 ± 0.019	0.074 ± 0.017	0.076 ± 0.012	0.084 ± 0.014	0.078 ± 0.012
CVR/HW (mm Hg min/ml/100 g)	0.184 ± 0.069	0.233 ± 0.057	0.243 ± 0.053	0.269 ± 0.071	0.248 ± 0.057
CVC (ml/min/mm Hg)	19.4 ± 7.2	14.3 ± 3.9	13.5 ± 2.7	12.3 ± 2.3	13.6 ± 2.5
CVC/HW (ml/min/100 g/mm Hg)	6.3 ± 2.5	4.6 ± 1.2	4.3 ± 1.0	4.0 ± 1.1	4.4 ± 1.0

AoP, aortic pressure, CBF, coronary blood flow; CVC, coronary vascular conductance; CVR, coronary vascular resistance; DCD, donation after circulatory death; HW, heart weight; MDO_2_, myocardial oxygen delivery; MEO_2_, myocardial oxygen extraction; MVO_2_, myocardial oxygen consumption.

Upon reperfusion, CVR was low (0.058 ± 0.019 mm Hg min/ml) but increased slightly within the ensuing hours on ESHP, whereas CVC decreased (Table 2). Median infusion rate of adenosine was 4.6 (1.6-9.9) ml/h which translates into a delivered dosage of 6.9 (2.4-14.9) µg/h.

### Correlations

**Figure 1:**
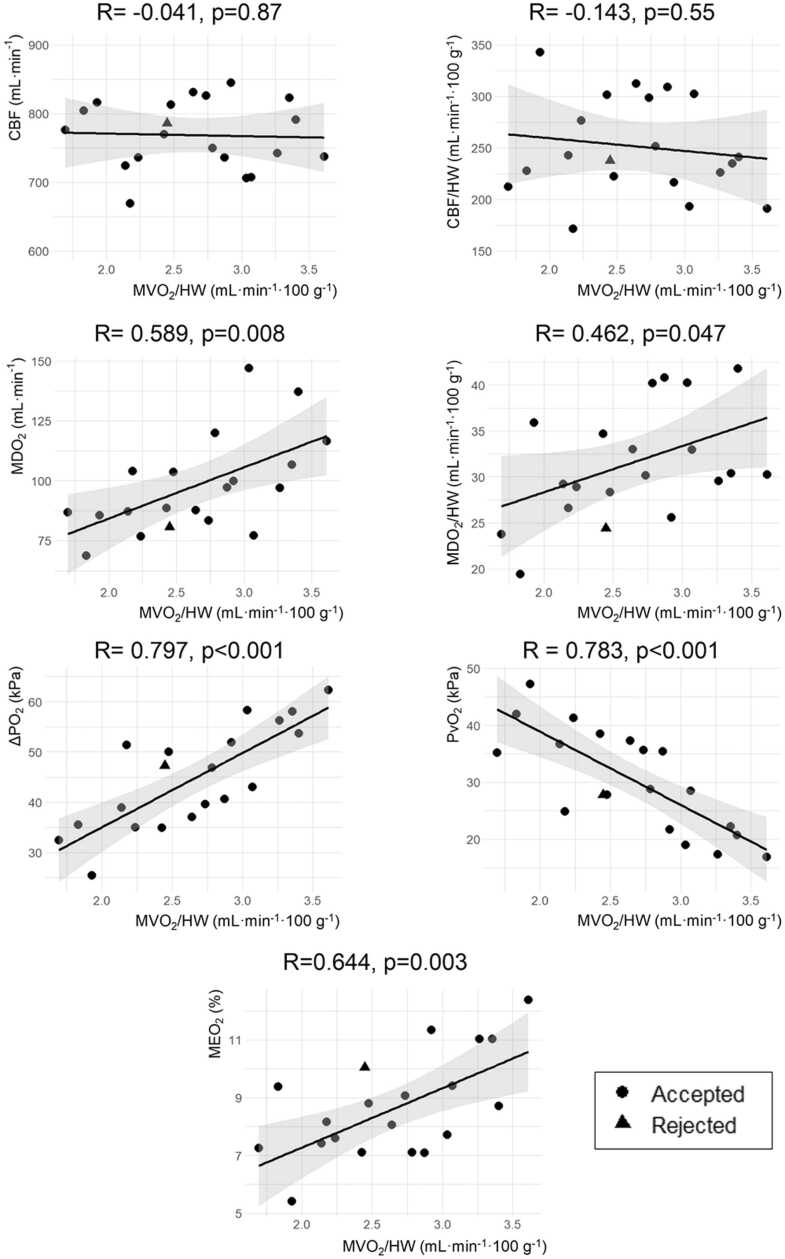
Determinants of myocardial oxygen consumption (MVO_2_). MVO_2_/HW did not correlate with coronary blood flow (CBF) and was moderately correlated to the delivery of oxygen (MDO_2_) and oxygen extraction (MEO_2_). In contrast, MVO_2_/HW strongly correlated with the arteriovenous difference in partial pressure of oxygen (Δ*P*O_2_) and venous partial pressure of oxygen (*P*vO_2_). Correlations were calculated for transplanted hearts (*n* = 19) and the rejected heart (*n* = 1) was plotted for comparison. HW, heart weight.

**Figure 2:**
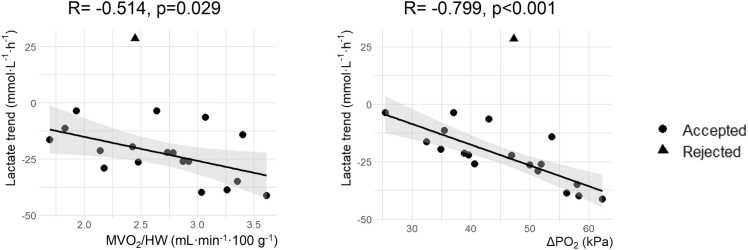
Correlations between the change in lactate levels over time and average myocardial oxygen consumption (MVO_2_/HW) and arteriovenous difference in partial pressure of oxygen (Δ*P*O_2_). Correlations were calculated for transplanted hearts (*n* = 19) and the rejected heart (*n* = 1) was plotted for comparison. HW, heart weight.

The average MVO_2_ showed moderate correlation with MDO_2_ (R = 0.589, *p* = 0.008) and MEO_2_ (R = 0.644, *p* = 0.003), but not with CBF (R = −0.041, *p* = 0.87) (Figure 1). Strong correlations were observed between MVO_2_ and Δ*P*O_2_ (R = 0.797, *p* < 0.001) and *P*vO_2_ (R = 0.783, *p* < 0.001) (Figure 1). In addition, the change in lactate over time of transplanted hearts showed strong inverse correlation with Δ*P*O_2_ (R = −0.799, *p* < 0.001) and moderate inverse correlation with MVO_2_ (R = −0.514, *p* = 0.029) (Figure 2). In other words, a higher Δ*P*O_2_ resulted in more negative lactate trends and higher MVO_2_.

When comparing the rejected heart to the transplanted hearts, its Δ*P*O_2_ (47.3 kPa), MVO_2_ (2.4 ml/min) and MEO_2_ (10.0%) were similar, despite the increasing lactate trend (Figure 2).

### Postoperative ECMO support and adenosine infusion

From the 25 transplantations, 23 patients (92%) were alive 30 days after transplantation. In total, 8 recipient patients (32%) required ECMO after transplantation. From these, 7 patients could be successfully weaned from ECMO, with a median ECMO duration of 5 (5-7) days.

**Figure 3:**
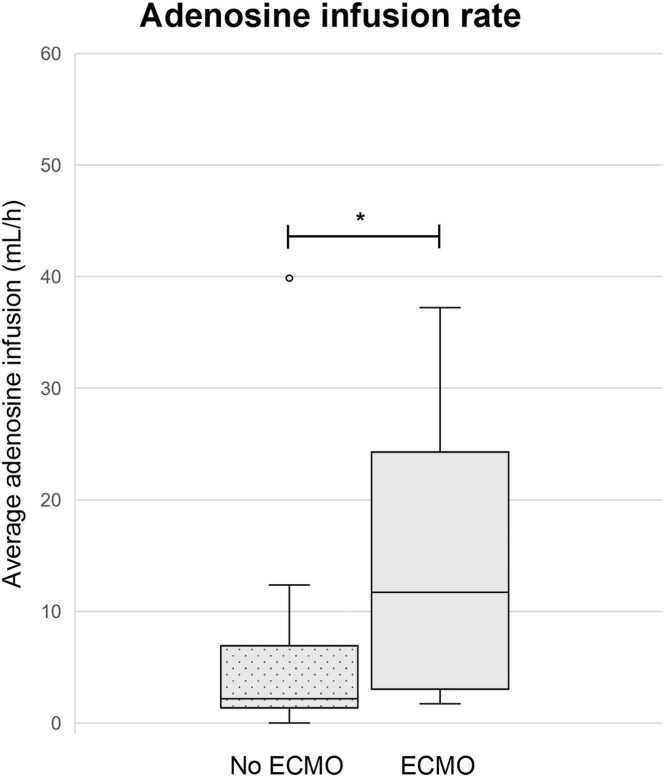
Average adenosine infusion rate on the OCS of patients that required (*n* = 8) vs patients that did not require (*n* = 17) ECMO support after transplantation. One outlier was present in the no ECMO group. Hearts from patients who required ECMO support after transplantation received significantly more adenosine on the OCS (*p = 0.039). ECMO, extracorporeal membrane oxygenation; OCS, Organ Care System.

None of the discussed oxygen handling parameters were significantly different between both groups, except for the median adenosine infusion rate (ECMO: 11.7 (4.5-21.0) ml/h vs no ECMO: 2.2 (1.5-6.7) ml/h, *p* = 0.039) (Figure 3). In other words, recipients of which the donor heart received more adenosine during ESHP, were more prone to require ECMO support post-transplantation. Yet, one heart that received a high amount of adenosine (39.8 ml/h) due to high AoPs did not require ECMO support (Figure 3). Median FWIT (ECMO: 16.3 ± 3.5 minutes vs no ECMO: 17.2 ± 4.2 minutes, *p* = 0.60) and time on OCS (ECMO: 257 ± 70 minutes vs no ECMO: 266 ± 62 minutes, *p* = 0.745) were not different between groups. In the ECMO group, 5 out of 8 patients (63%) were resternotomy cases compared to 14 out of 17 patients (82%) in the group not requiring ECMO support.

## Discussion

### Key findings

This is the first clinical study to evaluate the balance between MDO_2_ and MVO_2_ of human DCD hearts transported on normothermic ESHP for cardiac transplantation. Hearts on OCS are exposed to supraphysiological CBF rates containing very high oxygen concentrations, resulting in excessive MDO_2_. In contrast, MVO_2_ of DCD hearts on the OCS is relatively low and does not present as a strong marker of metabolic performance under these circumstances. The arteriovenous Δ*P*O_2_ could be a valuable additional parameter of metabolic performance. Furthermore, the average infusion rate of adenosine needed on the OCS could be an important risk factor for postoperative ECMO use.

### High myocardial oxygen delivery

In normal physiology, CBF is tightly coupled to balance MDO_2_ and MVO_2_. Additional oxygen can only be delivered through an increased CBF, which is under strict metabolic control of the coronary resistance vessels.^20^ On the OCS, MDO_2_ is very high compared to resting physiological values, due to a 6 to 7 times higher *P*aO_2_ (75.2 ± 2.9 kPa vs 10.5-13.5 kPa) and 2 to 3 times higher CBF (773 ± 52 ml/min vs 250-450 ml/min).^20^ However, only a minimal percentage of this available oxygen is consumed (MEO_2_: 8.6 ± 1.8%). Therefore, it seems superfluous to supply such an excessive quantity of oxygen, given that a large portion of the available oxygen remains unutilized.

On the OCS, the standard setting uses a gas mixture with 85% oxygen, 1% carbon dioxide and a nitrogen balance, with a default gas flow rate of 150 ml/min.^22^ The 1% carbon dioxide concentration in the gas mixture seems logical to balance pH, but the 4 times higher oxygen concentration, as compared to 21% atmospheric, is not supported by scientific literature. In fact, hyperoxia may be harmful for the donor heart, since it is known to cause oxidative stress in the myocardium.^25^, ^26^ In the setting of ischemia and reperfusion, oxidative stress due to the generation and accumulation of reactive oxygen species is inevitable, but hyperoxia could further aggravate cellular damage and ischemia-reperfusion injury. As such, hyperoxia could negatively affect outcomes of ESHP and subsequent transplantation as enhanced oxidative stress is responsible for myocardial function decline during ESHP.^8^, ^27^, ^28^ Furthermore, hyperoxia induces coronary vasoconstriction,^25^, ^29^, ^30^, ^31^ paradoxically leading to decreased tissue oxygenation.^30^, ^31^, ^32^ This might be the reason why CVR is increasing during ESHP in our study (Table 2), which is corroborated by observations from other studies.^33^ For this reason, guidelines recommend avoiding hyperoxia in patients after recovery from a cardiac arrest event,^34^ which should perhaps also be applied to the setting of DCD heart transplantation. Hence, to provide more physiological concentrations to the heart, the gas composition on the OCS should be changed to a mixture with lower oxygen concentrations.

Furthermore, CBF is relatively high on the OCS, because maintenance of cardiac function is dependent on adequate CBF. However, prevention of coronary overflow is equally important,^20^ since excessive CBF can induce endothelial damage and myocardial edema, which have been associated with primary graft dysfunction.^35^, ^36^, ^37^ Recent studies demonstrated that contractile dysfunction develops within several hours on ESHP^38^, ^39^ and regulation of coronary vasomotor tone is altered.^40^ In contrast, a lower [Hb] of 8.2 ± 1.9 g/dl during ESHP due to dilution of the donor blood with priming solution, compared to 12 to 18 g/dl in-vivo, argues for a higher CBF.^41^ However, it is undefined which CBF is appropriate to compensate for this deficiency. Experimental studies have shown adequate perfusion with CBF as low as 500 ml/min^37^, ^42^ and maintenance of cardiac energy stores with AoP as low as 40 mm Hg.^38^ Furthermore, flow-controlled coronary perfusion was demonstrated to be superior to pressure control in terms of myocardial contractile function and edema formation by Qi et al.^37^ Thus, to reduce the overall reported myocardial edema formation during ESHP, it could be considered to lower CBF to physiological values and tailor OCS settings to CBF rather than the “auto settings” of AoP, where safeguarding coronary autoregulation should be key. The MEO_2_ can potentially serve as an important marker to reduce CBF, because MEO_2_ is <10% on the OCS, compared to physiological MEO_2_ of 70% to 80%.^20^ MEO_2_ adaptation toward more physiological levels could guide safe reduction of CBF.

### Low myocardial oxygen consumption

The MVO_2_ of donor hearts on OCS is relatively low (2.7 ± 0.6 ml/min/100 g) compared to physiological resting values in human hearts (8-13 ml/min/100 g).^43^ This is probably caused by the absence of left ventricular loading during Langendorff perfusion on the OCS.^44^, ^45^, ^46^ Yaku et al showed that unloading of the left ventricle in Langendorff-perfused rabbit hearts reduced MVO_2_ from 6.0 to 2.7 ml O_2_/beat/g,^45^ and Gibbs et al showed that MVO_2_ drops from 9.2 to 3.8 ml/100 g/min when both ventricles are completely unloaded.^46^ Furthermore, temperature is slightly decreased on the OCS (34°C) compared to in-vivo (37°C), also reducing MVO_2_.^47^

Besides, low MVO_2_ rates might be characteristic of a heart procured with DCD. However, a direct comparison between the MVO_2_ of DCD and DBD hearts has not yet been reported in human hearts on OCS. Yet, ESHP studies from Ribeiro et al and White et al using porcine hearts did not find a difference in MVO_2_ between DBD and DCD hearts.^16^, ^17^

### Correlations between myocardial oxygen handling and CBF

The MVO_2_ did not correlate with CBF, although a direct linear relationship was expected based on Equation (5) and earlier studies.^20^, ^48^ This means that, in the range of reported CBF rates (650-850 ml/min), an increased CBF does not lead to increased MVO_2_. In current clinical practice, CBF is often increased upon increasing lactate trends,^22^ since it is assumed that an increasing lactate is the result of poor perfusion. However, our results suggest that increasing the CBF does not lead to increased MVO_2_, and might even hamper the autoregulatory coronary vasoreactivity.^35^ Furthermore, our results also suggest that decreasing the CBF does not decrease MVO_2_, which provides another argument to lower CBF on the OCS. Alternatively, an increase in MDO_2_ could be accomplished by increasing [Hb] in the perfusate (Equations (3) and (4)), which is possible by adding banked blood, however, at the expense of consequential citrate accumulation and increased lactate and potassium concentrations.^49^ Kobayashi et al. showed lower MVO_2_ and worse contractile function in hearts perfused with low [Hb].^50^

### Clinical assessment of metabolic function

The fact that the OCS is based on a Langendorff setup makes standard assessments of cardiac function impossible and the current assessment predominantly relies on lactate profiles. The OCS protocol dictates that hearts can be transplanted if lactate levels are decreasing and visual contractility is good.^22^ Yet, lactate profiles have been shown to be unreliable in predicting graft viability in up to one-third of procedures^5^ and several cases of increasing lactate trends with good postoperative outcomes have already been reported.^3^, ^11^, ^51^

Real-time MVO_2_ evaluation during ESHP could aid in predicting graft viability.^16^, ^17^ However, the standard formulas to calculate MVO_2_ might be less applicable when dealing with supraphysiological concentrations of oxygen in the perfusate (Equations (3) and (5)). The oxygen demand of the donor heart can be completely met with hemoglobin-unbound oxygen due to very high *P*aO_2_ (venous SO_2_ is often 100%) so MVO_2_ is only determined by Δ*P*O_2_. Therefore, Δ*P*O_2_ can be an additional marker of metabolic function on the OCS (under the current hyperoxic conditions), which is confirmed by the strong correlation of MVO_2_ with Δ*P*O_2_ (Figure 1) and lactate over time (Figure 2). A higher MVO_2_ probably resulted in more uptake of lactate from the perfusate, which is a preferred energy substrate in the normal heart.^52^

The Δ*P*O_2_ can be especially helpful in case of an increasing lactate trend during ESHP. For example, the rejected heart with an increasing lactate trend in our series showed no outliers in any of the presented correlations (Figures 1 and 2), including Δ*P*O_2_, which could suggest this heart got wrongfully rejected. We propose that Δ*P*O_2_ can serve as an additional parameter to assess metabolic function, especially in hearts with an increasing lactate trend that are currently rejected. Future studies should investigate the predictive value of Δ*P*O_2_ in such cases, which could eventually enable more transplantations. Remarkably, there was no significant arteriovenous difference in the *P*CO_2_, although a higher venous *P*CO_2_ would be expected in case of aerobic metabolism.^20^

### Coronary vascular resistance and evaluation of adenosine infusion

Adenosine exerts various effects on the heart and poses a contradiction in the ESHP setting. It is infused on the OCS for coronary vasodilation^53^, ^54^ upon low CBF with high diastolic AoP. In addition, adenosine increases contractile performance,^55^ promotes tissue protection and repair,^56^, ^57^, ^58^ decreases infarct size,^57^, ^59^ and decreases ischemia-reperfusion injury^60^, ^61^, ^62^, ^63^, ^64^, ^65^, ^66^ by inhibition of reactive oxygen species release.^58^ On the other hand, it is known to increase vascular permeability, which could contribute to the development of myocardial edema.^67^ Moreover, it seems counterintuitive to administer adenosine instead of honoring the normal autoregulatory function of the coronaries. Given the presented supraphysiological MDO_2_ settings combined with the fact that myocardial edema develops over time on the OCS, infusion of adenosine thus might try to solve a problem that could partially be prevented, thereby leaving coronary autoregulation intact. In this respect, it is important to know whether coronary autoregulation is disturbed due to warm ischemic injury. However, several studies have shown that following brief periods of myocardial ischemia which can produce myocardial stunning, coronary autoregulation remains intact.^68^, ^69^, ^70^ The observation that contractile function was preserved in all transplanted hearts (indicating negligible myocardial stunning) provides further reassurance that coronary autoregulation is preserved.^71^

In our study, recipients of a donor heart that received more adenosine on the OCS had a higher rate of ECMO implantations. This is corroborated by the Papworth group,^5^ who already suggested high adenosine infusion requirement as a criterion to consider rejection of the heart. Since it is unclear whether the need for ECMO is a cause or an effect of adenosine, its precise role in the OCS requires further investigation. Either these hearts might have less coronary reserve resulting in higher perfusion pressures with lower CBF, or the higher rate of ECMO implantations might be partially contributed to deleterious effects of adenosine itself. In our experience, high adenosine infusion above standard settings, does not always lead to an additional increase in CBF. Therefore, we suggest to be careful with high rates of adenosine infusion during EVHP.

Furthermore, adenosine is mixed in a maintenance solution, so an increase in its infusion rate also results in increased infusion of other substances such as glucose, often resulting in hyperglycemia on the OCS.

### Limitations

The current study investigated oxygen handling and coronary vasoreactivity of human DCD hearts on the OCS, but did not investigate DBD procedures since these are not performed using the OCS in the Netherlands. The present retrospective study included 25 DCD hearts transplanted in a single center. It is likely that our findings will be similar to other centers as we used the standard OCS protocol, yet confirmation in a larger multicenter study is important. Additionally, the retrospective nature of our study was associated with missing blood gas data, and future prospective studies should minimize the occurrence of missing data. The current study did not account for recipient characteristics, which is also influencing the need for ECMO support post-transplantation. In addition, histological analyses were not performed on the rejected heart, so the cause of increasing lactate concentrations is unknown. Lastly, this study used statistical correlation analyses, thus was only able to demonstrate associations, no causal relations.

## Conclusions

A supraphysiological concentration of oxygen is delivered to donor hearts on the OCS, which might paradoxically increase ischemia-reperfusion injury by increased coronary vasoconstriction. Together with the high CBF, this might lead to additional myocardial edema formation. In fact, we showed that hearts on OCS have a relatively low and stable MVO_2_ mostly using hemoglobin-unbound oxygen in the blood, emphasizing the excess of oxygen as well as CBF in this system. In light of these observations, a reduction of *P*O_2_ and CBF during normothermic ESHP could be considered. Furthermore, under the current hyperoxic preservation conditions, Δ*P*O_2_ can serve as an additional marker of metabolic function to aid in graft assessment on the OCS, and potentially increase the acceptance rate of hearts for transplantation. Finally, the increased infusion of adenosine on the EVHP system could serve as a potential predictor for postoperative ECMO.

## Author contributions

Jorik H. Amesz: Conceptualization, Methodology, Formal analysis, Investigation, Data curation, Writing—Original draft, Writing—Review and editing, Visualization. Sanne J.J. Langmuur: Formal analysis, Investigation, Data curation, Writing—Original draft, Writing—Review and editing, Visualization. Mark F.A. Bierhuizen: Formal analysis, Investigation, Data curation, Writing—Original draft, Writing—Review and editing. Dwight Dumay: Resources, Data curation, Writing—Review and editing. Pieter C. van de Woestijne: Resources, Writing—Review and editing. Jelena Sjatskig: Resources, Writing—Review and editing. Lisa E. Sluijter: Resources, Data curation, Writing—Review and editing. Dirk J. Duncker: Validation, Writing—Review and editing. Olivier C. Manintveld: Resources, Data curation, Writing—Review and editing, Supervision. Yannick J.H.J. Taverne: Conceptualization, Methodology, Resources, Writing—Original draft, Writing—Review and editing, Supervision.

## Disclosure statement

The authors declare that they have no known competing financial interests or personal relationships that could have appeared to influence the work reported in this paper. O.C. Manintveld reports a relationship with Abbott, Astra Zeneca, Boehringer Ingelheim, Daiichi-Sankyo, Novartis, Novo Nordisk, and Siemens that includes speaking and lecture fees. O.C. Manintveld reports a relationship with Cardiovasculair Onderwijsinstituut (CVOI) Nederland, and Dutch Cardiac Society Working Group Heart Failure that includes board membership. The other authors declare that they have no known competing financial interests or personal relationships that could have appeared to influence the work reported in this paper.
